# The c-Raf modulator RRD-251 enhances nuclear c-Raf/GSK-3/VDR axis signaling and augments 1,25-dihydroxyvitamin D3-induced differentiation of HL-60 myeloblastic leukemia cells

**DOI:** 10.18632/oncotarget.24275

**Published:** 2018-01-19

**Authors:** Harrison T. Supnick, Rodica P. Bunaciu, Andrew Yen

**Affiliations:** ^1^ Department of Biomedical Sciences, Cornell University, Ithaca, NY 14853, USA; ^2^ University of Pittsburgh School of Medicine, Pittsburgh, PA 15261, USA

**Keywords:** RRD-251, myeloid, HL-60, MAPK, GSK-3

## Abstract

Differentiation therapy is used in cancer treatment. Epidemiologic studies showed that higher vitamin D levels are associated with reduced cancer risks. However, the therapeutic doses needed for differentiation are accompanied by hypercalcemia and intolerable pathological sequelae. In the present work we evaluated if RRD-251, a small-molecule, can enhance vitamin D3-induced differentiation of leukemic cells, in the hope of decreasing the needed vitamin D3-dose.

We demonstrate that RRD-251 enhances vitamin D3-induced differentiation of leukemic cells, the enrichment of the c-Raf kinase in the nucleus, the binding of nuclear c-Raf to GSK-3, increased phosphorylation of GSK-3 ser 21/9 inhibitory sites, and the binding of GSK-3 to VDR, where GSK-3 inhibition is known to enhance transcriptional activation by VDR. Enhancement of D3-induced p-GSK-3 ser 21/9 by RRD-251 was associated with enhanced Akt-GSK-3 binding, Akt being a known GSK-3 inhibitor, and diminished Erk1/2 binding. Diminishing Erk interaction with GSK-3 was associated with enhanced interaction with Vav1, a known driver of myeloid differentiation. This is redolent of an ATRA/c-Raf/GSK-3/RARα axis we previously reported, although the phosphorylation effects to enhance transcriptional activation on RARα vs VDR diverge. Taken together this indicates potential therapeutic significance for a c-Raf/GSK-3/VDR or RARα axis for leukemic myelo-monocytic differentiation.

## INTRODUCTION

While acute myeloid leukemia (AML) is the most common acute leukemia in adults, standard chemotherapies result in poor patient outcomes [1]. Despite the 60-80% of patients who are able to achieve remission, most relapse with treatment-resistant cancers within 2-3 years, and 5-year survival rates remain as low as 30% [2–4]. The failure of traditional chemotherapies in AML is believed to be, in part, due to their inability to address the underlying pathophysiology of AML, a maturation arrest where non-terminally differentiated myeloid precursor cells proliferate unchecked [5, 6].

All-trans-retinoic acid (RA) therapy addresses the blast maturation arrest by inducing myeloid differentiation in acute promyelocytic leukemia (APL), revolutionizing the treatment of this rare subtype of AML [7]. Under contemporary regimens of RA and arsenic trioxide, APL has been transformed from one of the most deadly to the most treatable myeloid leukemias with cure rates as high as 80-90%. Since RA therapy has lower toxicity compared to traditional chemotherapies, and lacks the emergence of secondary tumors, tremendous interest lies in extending its applicability to AML [6, 8]. Unfortunately, RA alone does not induce differentiation categorically in AML, and only inhibits proliferation or induces apoptosis [9]. Similar to RA, 1,25-dihydroxyvitamin D3 (D3) is considered a promising differentiation agent for AML [10]. Indeed, in HL-60 cells, lineage bipotent acute myeloblastic cells (a non-APL, NCI-60 reference cell line), RA induces myeloid differentiation, whereas D3 induces monocytic differentiation, both with prominent cell cycle arrest [11, 12]. Despite promising effects on *in vitro* differentiation, in clinical trials therapeutic doses of D3 resulted in unacceptably high levels of side effects resulting from hypercalcemia. Thus, characterizing novel differentiation-promoting agents that are able to lower the effective dosage of D3, and prevent hypercalcemia, may allow for the adoption of D3 as a viable differentiation therapy. Combination differentiation therapy may thus be better than using a single agent.

Reflecting an early common lineage non-specific priming that occurs during RA/D3-induced differentiation, a common ensemble of proteins forms shared RA/D3 differentiation machinery in HL-60 cells. Durable signaling through the MAPK pathway is transmitted through c-Raf (MAPKKK) which drives differentiation [13–15]. While in the traditional transient MAPK signaling pathway c-Raf activates Mek which promotes Erk regulation of nuclear transcription factors, in the prolonged RA/D3 associated signaling, c-Raf additionally translocates to the nucleus by 48 hours to activate its own transcription factor targets [16, 17]. One such transcription factor is NFATc3 which promotes transcription of BLR1/CXCR5, a receptor necessary for driving differentiation, an effect inhibited by PD98059 [18]. Consistent with this, overexpression of c-Raf enhances MAPK and BLR1 signaling and increases differentiation while inhibition blocks interactions with transcription factors and ultimately differentiation [13]. The enhancement of c-Raf signaling indicates that there are important roles and novel targets for c-Raf other than Mek for driving differentiation. Another target of c-Raf in the nucleus, the Retinoblastoma protein (RB) is another key regulator of the cell cycle and thus differentiation in HL-60 cells [19, 20]. The progression of the cell cycle is marked by progressive RB hyperphosphorylation; conversely, the hypophosphorylation of RB is associated with G_0_ cell cycle arrest, and is seen most significantly by 72 hours post RA/D3 treatment. Hypophosphorylated RB sequesters E2F transcription factors to prevent expression of genes necessary for entering S-phase [21]. RA-induced RB-E2F complexes begin to appear at 48 hours and are inhibited by phosphorylation of RB at serine 608 [22]. This same phosphorylation site shows a transient interaction with c-Raf early in the differentiation time course, which concomitantly acts as a reservoir, releasing nuclear c-Raf to interact with other partners during later differentiation events as RB hypophosphorylates [23].

Recent studies indicate that inhibition of glycogen synthase kinase-3 (GSK-3) can induce differentiation in AML cells on its own [24, 25] and enhance RA and D3 differentiation therapies via respective hyperactivation of RARα and VDR transcriptional activity [23, 26]. Interestingly, the mechanisms of transcriptional hyperactivation differ. Inhibition of GSK-3 leads to hypophosphorylation of RARα which relieves the inhibition of RARα transcriptional activation. Counterintuitively, GSK-3 inhibition leads to hyperphosphorylation of VDR with the same effect of enhanced transcriptional activation [26]. GSK-3 directly binds RARα and modulates its transcriptional activity, but seems to affect VDR indirectly via intermediates such as the coactivaror NCOA3 [26]. Hence while there are commonalities there are also fundamental differences between RARα and VDR regulation by GSK-3. Additionally, GSK-3 inhibitors demonstrate acceptable toxicity on their own and importantly, sensitize AML response to lower, less toxic concentrations of D3 [27].

Initially synthesized by Chellappan and colleagues, RRD-251, a selective disrupter of the interaction between c-Raf and RB, enhances RA-induced myeloid differentiation of HL-60 cells through a novel c-Raf/GSK-3/RARα axis [23, 28]. Dissociation of c-Raf and RB leads to increased E2F sequestration by RB and G_1_/_0_ arrest, while simultaneously increasing associations of c-Raf with other nuclear partners including GSK-3. Furthermore, RRD-251 amplifies both the RA-induced nuclear serine 21/9 inhibitory phosphorylation of GSK-3 and suppresses GSK-3’s inhibition of RARα, leading to increased RARα transcriptional activity as seen by increased RARE-driven CD38 expression.

A basic question being addressed here is whether the c-Raf/GSK-3 axis is conserved for both myeloid and monocytic differentiation; ie., given that it is used by RA for RARα is it also used by D3 for VDR. Mechanistically, if c-Raf nuclear translocation is important to monocytic differentiation, then the GSK-3 regulation of VDR implies that RRD-251 should promote D3 induced differentiation. And we show that this is the case – namely that the nuclear c-Raf promotes D3 action if cells are presented with D3 and RA action if cells are presented with RA. We confirm the putative c-Raf/GSK-3 axis as a conserved component in the enhancement of differentiation. Paralleling RA, combined treatments of RRD-251 with D3 lead to increased interactions with c-Raf and amplified nuclear serine 21/9 inhibitory phosphorylation of GSK-3. Our next aim was to better characterize these interactions, observing that total c-Raf and the 259 and 621 serine-phosphorylated species demonstrated increased nuclear interactions with both total GSK-3 and the serine 21/9 inhibitory phosphorylated GSK-3. Additionally, we noted the convergence of multiple kinase signaling pathways on serine 21/9 inhibitory phosphorylation of GSK-3, namely an enhancement of a D3-induced, nuclear Akt interaction with GSK-3. As with combined RA and RRD-251 treatments, D3 co-treatments further decreased nuclear GSK-3 interactions with Erk, promoting downstream increased interactions between Erk and Vav1. Vav1 plays an integral role in differentiation as studies that prevent Erk from interacting with Vav1 inhibit differentiation by eliminating Vav1’s binding and activation of the CD11b promoter [29, 30]; therefore an increased interaction of these binding partners may drive the observed increases in CD11b expression. Downstream of the enhanced GSK-3 inhibition, we also report novel, increased interactions of GSK-3 with the VDR and decreased interactions with RXRα. As GSK-3 inhibitors lead to hyperphosphorylation and hyperactivation of the VDR, the direct binding with GSK-3 suggests a role in directly activating the VDR to enhance D3-driven differentiation. Coincidentally, the decreased interaction between GSK-3 and RXRα may be important for enhanced heterodimerization with the VDR to further amplify transcriptional activation at VDREs. Our results indicate a novel D3/c-Raf/GSK-3/VDR axis that drives VDR transcriptional hyperactivation and enhances differentiation under conditions that stimulate endogenous GSK-3 inhibitory phosphorylation. Activation of the novel D3/c-Raf/GSK-3/VDR axis is propelled by RRD-251 regulation with the end result being enhanced blast differentiation. These results suggest an increased potential for use of RRD-251 in combination therapies in AML cells and support a new mechanism contributing to D3-induced differentiation.

## RESULTS

### RRD-251 enhances D3-induced differentiation

Motivated by the enhancement of RA-induced differentiation in HL-60 cells [23], we sought to determine if co-treating with RRD-251 had a similar impact on D3-induced differentiation. Like RA, D3 induces phenotypic changes in growth arrest, inducible oxidative metabolism, and the surface markers CD38 and CD11b, as well as CD14, a marker specific to the monocyte lineage. As previously reported, RRD-251 treatment alone induces cell cycle retardation [23]. However, in combination with D3, the expression of each of these markers was augmented over cells treated with only D3.

**Figure 1:**
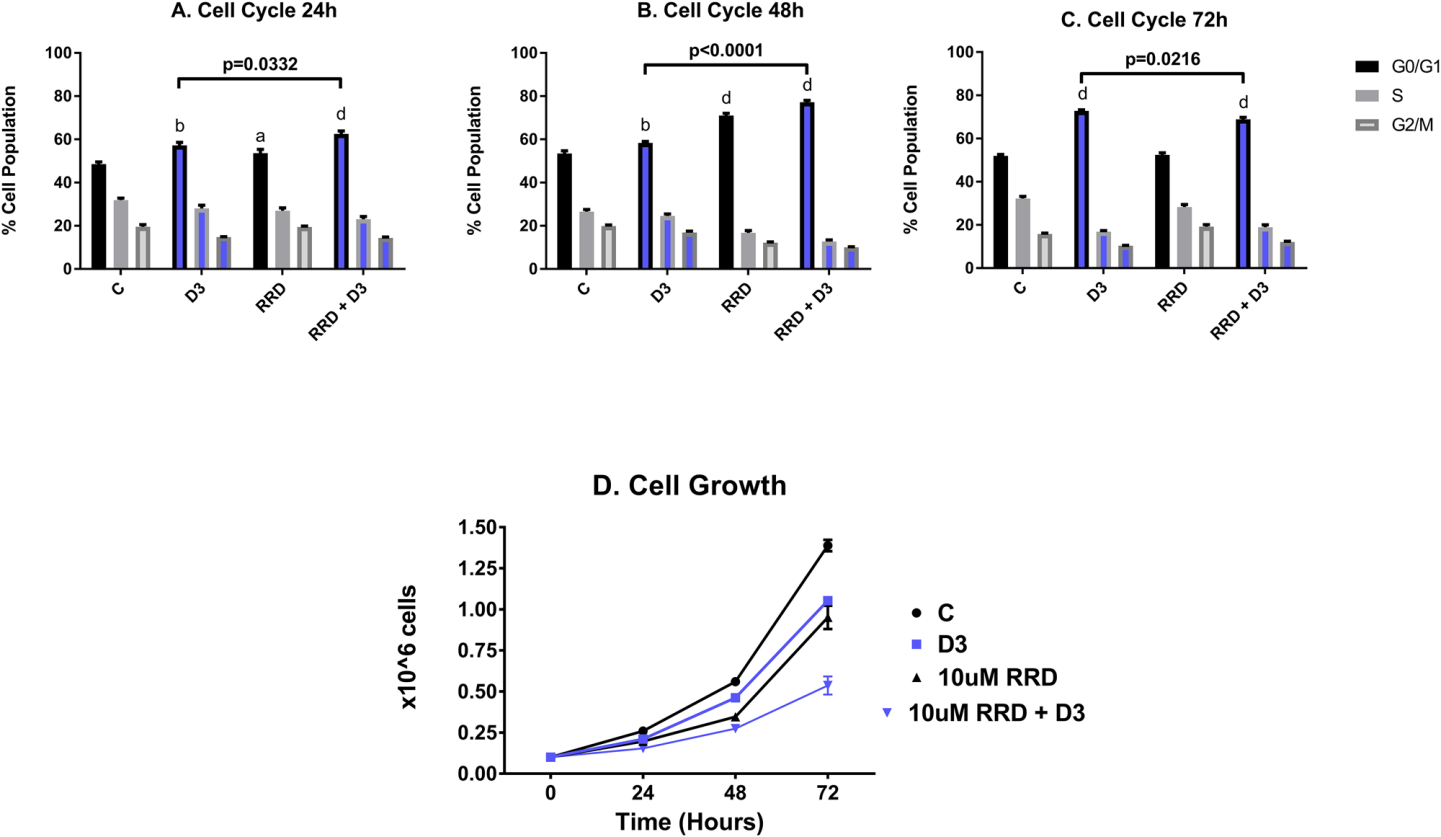
RRD-251 enhances D3-induced G_1/0_ cell cycle arrest and inhibition of proliferation HL-60 cells were untreated (C) or treated with 0.5 μM D3, 10 μM RRD-251, or D3 plus 10 μM RRD-251 for each designated time point. Various levels of significant change vs control are denoted by p< a-d, where a=0.05, b=0.01, c=0.001, d=0.0001. Statistically significant changes of G_1/0_ (vs. control) are labeled a, b, d on the graphs and significant changes between D3 and RRD-251 plus D3 are highlighted. C is untreated control, D3 is D3 treated, RRD is RRD-251 treated, RRD + D3 is RRD-251 plus D3 treated cultures. **(A)** Cell cycle analysis at 24 hours, as measured by flow cytometry with propidium iodide staining, indicates that cell cycle distribution displayed a significant enrichment in G_1/0_ for cells treated with D3 or RRD-251 alone which is significantly by co-treatment (C vs. D3 p=0.0042; C vs RRD-251 p=0.0408; C vs. RRD-251 + D3 p=0.0004; D3 vs RRD-251 + D3 p=0.0332). **(B)** At 48 hours post treatment, enrichment in G_1/0_ increases for all treatments, and combined RRD-251 plus D3 cell cycle arrest continues to be significantly greater than for D3 alone (C vs. D3 p=0.0023; C vs. RRD-251 p<0.0001, C vs. RRD-251 + D3 p<0.0001; D3 vs. RRD-251 + D3 p<0.0001). **(C)** At 72 hours, cell cycle distributions became more similar for D3 vs D3 plus RRD-251, and D3-induced G_1/0_ enrichment slightly exceeds the combined treatment (C vs. D3 p<0.0001; C vs. RRD-251 p=0.7040, C vs. RRD-251 + D3 p<0.0001; D3 vs. RRD-251 + D3 p=0.0216) possibly reflecting a small partial synchronization effect. **(D)** As a measure of cell proliferation, cell densities were measured for both untreated and treated cells at 0, 24, 48, and 72 hours. D3 alone and RRD-252 alone each significantly reduced cell growth, and combined treatment showed further reductions (C vs. D3 p=0.0019; C vs. RRD-251 p=0.0005; C vs. RRD-251 + D3 p<0.0001; D3 vs. RRD-251 + D3 p=0.0002).

For all phenotyping experiments, HL-60 cells were untreated (C), or treated with D3, RRD-251, or D3 together with RRD-251. As immature blasts become terminally differentiated with D3 treatment, they lose the ability to proliferate and self-renew. At 24 hours after treatment, onset of cell cycle arrest was slightly enhanced for D3+RRD-251compared to treatment with D3 alone (C 48.57% G_1_/_0_, D3 57.23% G_1_/_0_ and RRD-251 + D3 62.57% G_1_/_0_) (Figure 1A). However, at 48 hours after treatment, RRD-251 treated cells exhibited greater G_0_/G_1_ enrichment than those treated solely with D3 (D3 58.43% G_1_/_0_ vs RRD-251 + D3 77.23% G_1_/_0_) (Figure 1B). At 72 hours after treatment, cell cycle arrest became similar for D3 and D3+RRD-251 -treated cells (C 51.97% G_1_/_0_, D3 72.77% G_1_/_0_ and RRD-251 + D3 68.9% G_1_/_0_) (Figure 1C). Cell population growth curves corroborated enhanced cell cycle arrest with RRD-251 augmenting D3-induced decreases in cell density (72 hours D3 vs RRD-251 + D3 p=0.0002) (Figure 1D). While both D3 and RRD-251 treatments alone significantly inhibited cell growth to a similar degree, D3 combined with RRD-251 significantly lowered the cell densities even further (1.388 x 10^6^ cells/mL for C, 1.053 x 10^6^ cells/mL for D3, 0.952 x 10^6^ cell/mL for RRD-251, 0.537 x 10^6^ cells/mL for RRD-251 + D3 at 72 hours after treatment). This is in agreement with enrichment in G_1_/_0_ at 48h.

**Figure 2:**
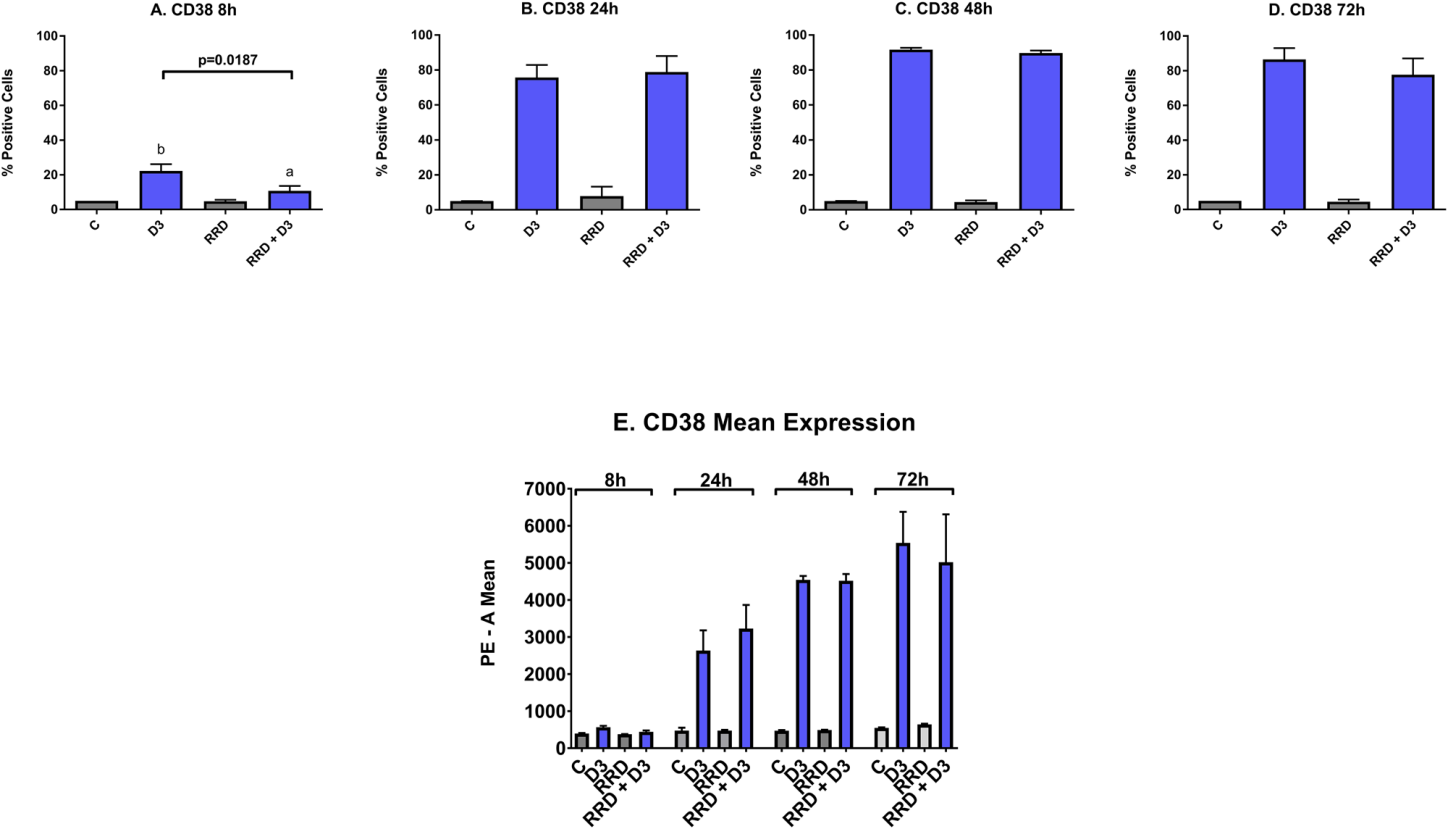
Effect of RRD-251 on CD38 HL-60 cells were untreated (C) or treated with 0.5 μM D3, 10 μM RRD-251, or D3 plus 10 μM RRD-251 for each designated time point. Statistically significant changes are labeled a and b on the graphs and significant changes between D3 and RRD-251 plus D3 are highlighted. **(A)** CD38 expression (assessed by flow cytometry with a PE-conjugated antibody) at 8 hours after treatment is induced by D3, but inhibited by RRD-251 (C vs. D3 p= 0.0030; D3 vs. RRD-251 + D3 p=0.0187). **(B)** CD38 expression at 24 hours post treatment is no longer inhibited by RRD-251 (D3 vs. RRD-251 + D3 p=0.6307). **(C)** At 48 hours, CD38 expression is nearly 100% and is not inhibited by RRD-251. **(D)** At 72 hours post treatment, CD38 remains close to 100% and is not inhibited by RRD-251. **(E)** At 24 hours post treatment, D3-induced CD38 mean expression per cell increases with RRD-251 although not to a significant degree (D3 vs. RRD-251 p=0.2951).

CD38 is an ectoenzyme receptor necessary for proper immune function [31–35], whose expression increases with D3 [36] and potentiates cellular signaling seminal to differentiation [37, 38]. Expression of CD38 is an early marker of differentiation. At the 8 hour time point, the increase in CD38 induced by D3 is delayed by RRD-251 (Figure 2A). The minute delay in the induction of CD38 expression due to RRD-251 is offset by increase in CD38 mean expression that occurs by 24 hours (Figure 2E). From 24 to 72 hours after treatment, the percentage of cells positively expressing CD38 is nearly 100% for both D3 and D3+RRD-251 (Figure 2B-2D).

**Figure 3:**
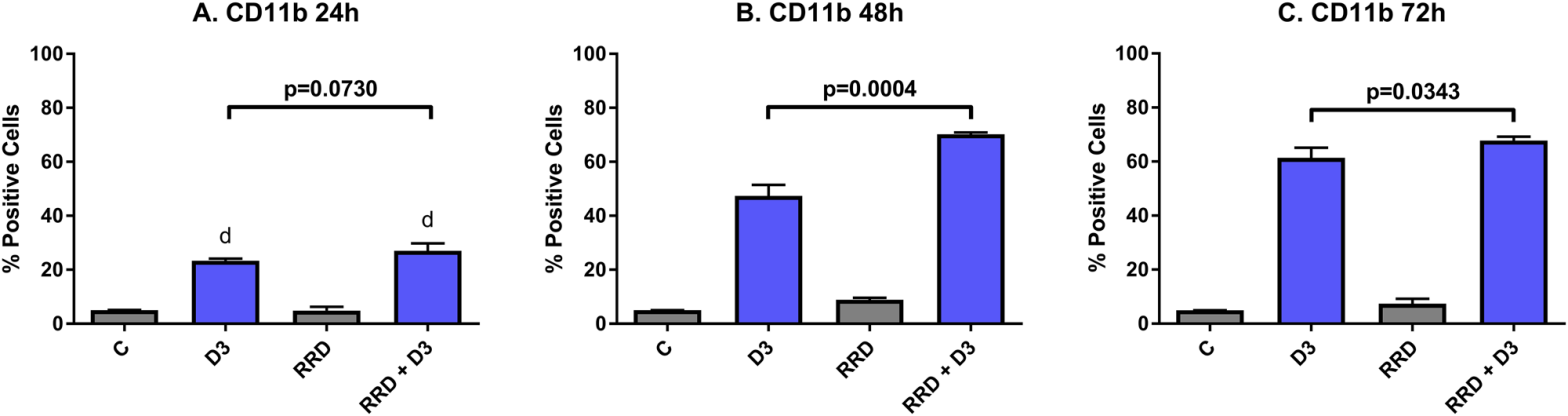
RRD-251 augments D3-induced CD11b expression HL-60 cells were untreated (C) or treated with 0.5 μM D3, 10 μM RRD-251, or D3 plus 10 μM RRD-251 for each designated time point. Statistically significant changes at 24 h of treated vs control samples are labeled d on the graph and significant changes between D3 and RRD-251 plus D3 are highlighted. **(A)** CD11b expression (assessed by flow cytometry with an APC-conjugated antibody) at 24 hours post treatment is increased by D3 treatment and augmented with combined RRD-251 treatment to just below the threshold for significance (C vs. D3 p<0.0001; C vs. RRD-251 + D3 p<0.0001, D3 vs. RRD-251 + D3 p=0.0730). **(B)** At 48 hours, D3-induced CD11b becomes significantly increased with the addition of RRD-251 (D3 vs. RRD-251 + D3 p=0.0004). **(C)** At 72 hours, D3-induced CD11b remains increased with RRD-251 co-treatment (D3 vs RRD-251 + D3 p=0.0343).

The late differentiation marker CD11b is the integrin αM subunit of the α_M_β_2_ heterodimeric integrin or complement receptor 3. Activation of CD11b thus activates the maturation of innate immune cells like granulocytes and monocytes and then allows for adhesion during leukocyte extravasation. D3 induces expression of CD11b by 24 hours post-treatment, and the early expression is similar with RRD-251 co-treatment (C vs D3 p<0.0001) (Figure 3A). But by 48 hours, D3-induced CD11b expression is augmented by RRD-251 (D3 vs RRD-251 + D3 p=0.0004; D3 47.33% positive and RRD-251 + D3 70.23% positive) (Figure 3B). At 72 hours, the trend of RRD-251 co-treatment enhancing D3-induced CD11b expression persists (D3 vs RRD-251 + D3 p=0.0343; D3 61.23% positive and RRD-251 + D3 67.77% positive) (Figure 3C).

**Figure 4:**
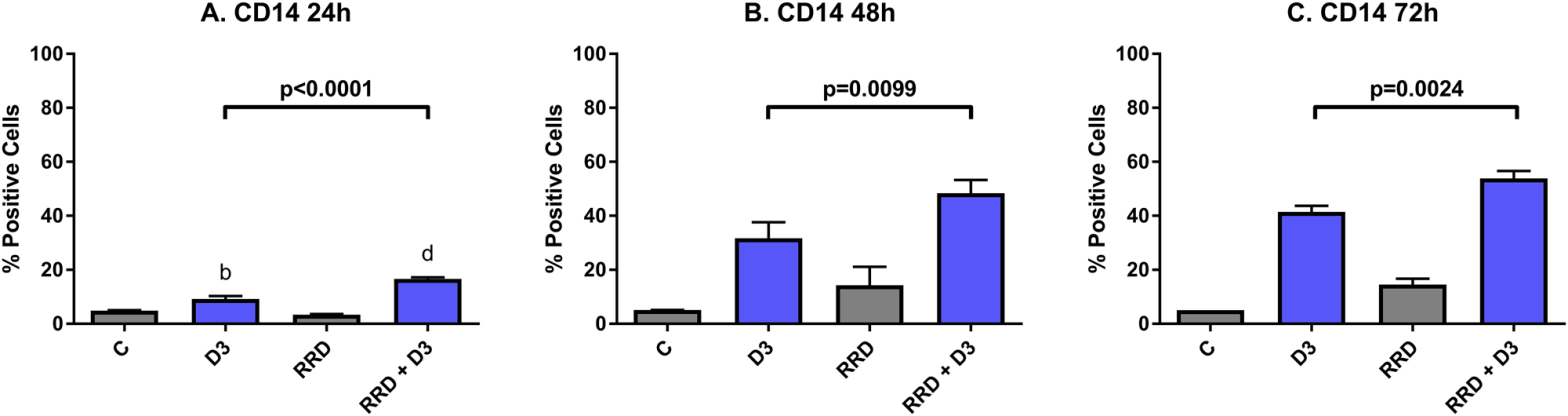
RRD-251 enhances D3-induced CD14 expression HL-60 cells were untreated (C) or treated with 0.5 μM D3, 10 μM RRD-251, or D3 plus 10 μM RRD-251 for each designated time point. Statistically significant changes are labeled b and d on the graphs and significant changes between D3 and RRD-251 plus D3 are highlighted. **(A)** CD14 expression (assessed by flow cytometry with a PE-conjugated antibody) at 24 hours post treatment is increased by D3 and enhanced by RRD-251 co-treatment (C vs. D3 p=0.0017; C vs. RRD-251 + D3 p<0.0001; D3 vs. RRD-251 + D3 p<0.0001). **(B)** At 48 hours post treatment RRD-251 continues to augment D3-induced CD14 expression (D3 vs. RRD-251 + D3 p=0.0099). **(C)** D3-induced CD14 expression remains increased with addition of RRD-251 at 72 hours post treatment (D3 vs. RRD-251 + D3 p=0.0024).

CD14 is a plasma membrane toll-like receptor that is activated by pathogen-associated molecular patterns, and is a monocyte lineage-specific late differentiation marker. At 24 hours post-treatment, D3-induced expression of CD14 is not yet great (9.17% positive), but co-treatment with RRD-251 (16.60% positive) significantly enhances CD14 expression (C vs. D3 p=0.0017; C vs. RRD-251 + D3 p<0.0001) (Figure 4A). By 48 hours, D3-induced CD14 expression increases and continues to be enhanced by RRD-251 (D3 vs. RRD-251 + D3 p=0.0099) (Figure 4B). At 72 hours, the trend continues that co-treatment enhances D3-induced CD14 expression (D3 vs RRD-251 + D3 p=0.0024) (Figure 4C).

**Figure 5:**
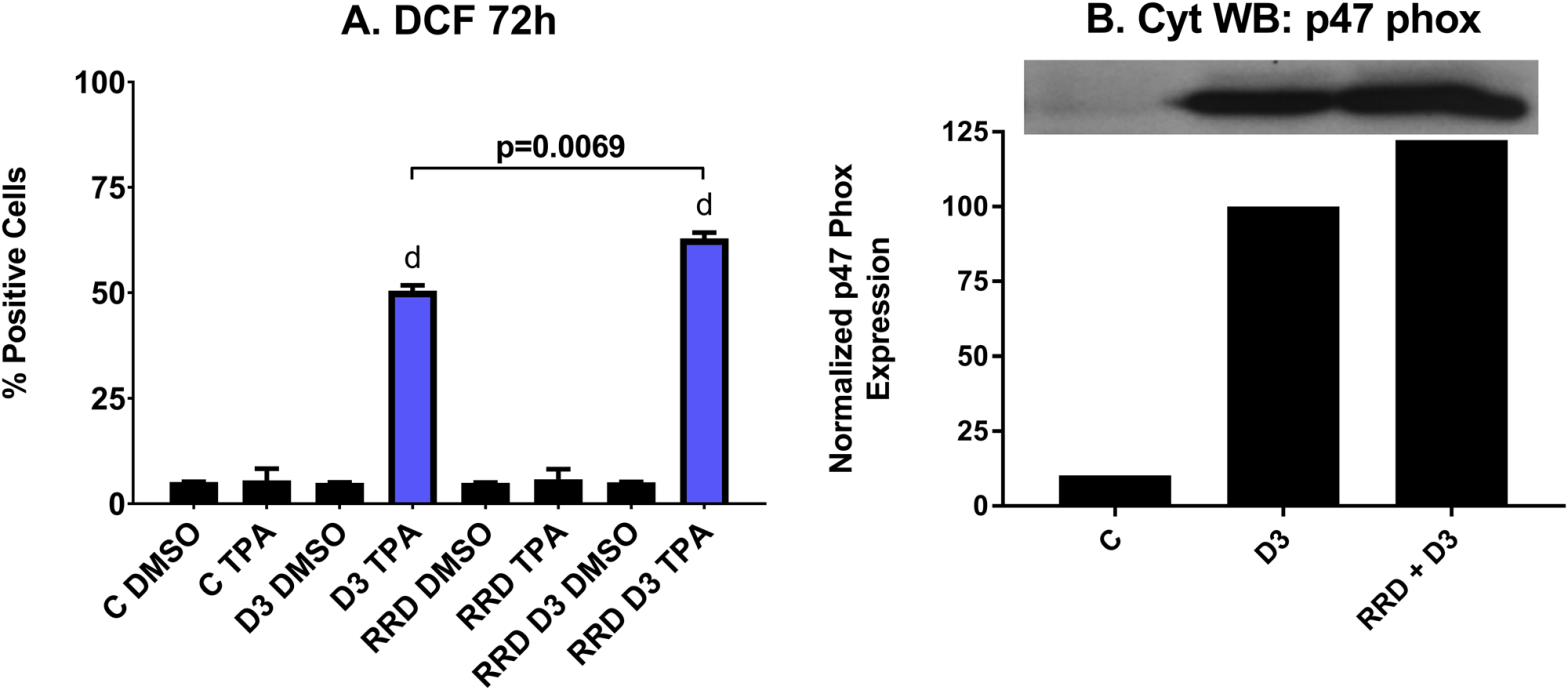
RRD-251 increases the D3-induced respiratory burst and oxidative metabolism **(A)** Respiratory burst from inducible oxidative metabolism, measured by flow cytometry of DCF stained cells at 72 hours post treatment, is significantly enhanced with combined RRD-251 plus D3 treatment over D3 alone (D3 vs. RRD-251 + D3 p=0.0069) C DMSO is D3 untreated control where DMSO is the control carrier blank for TPA, C TPA is D3 untreated control cells with the ROS inducible oxidative metabolism inducer, D3 DMSO is D3 treated but ROS measured with only the carrier blank sans TPA, D3 TPA is D3 treated and TPA induced, RRD indicates the parallel cases for RRD-251 treated cells, and RRD D3 indicates the parallel cases for RRD-251 plus D3 treated cells. **(B)** Expression of p47^phox^ a subunit of NADPH Oxidase was measured by Western blotting at 48 hours for untreated control, and D3 and RRD-251 plus D3 treated cells. RRD-251 significantly increases the D3-induced expression of p47^phox^. All the experiments were performed in triplicate (three biological repeats).

Reactive oxygen species (ROS) production by the NADPH oxidase complex kills bacteria ingested into phagolysosomes and is a functional marker of both granulocytes and monocytes. As a late differentiation marker for functionally differentiated and mature cells, ROS production is most apparent with D3 treatment after 72 hours. At 72 hours, just over 50% of cells treated with D3 alone are positive for ROS production, while over 60% of cells are positive when co-treated with RRD-251 (p=0.0069) (Figure 5A). As the NADPH oxidase complex is responsible for generating the ROS in the respiratory burst, cytosolic expression of p47^phox^, a subunit of the complex is an additional marker of functional differentiation. Indeed, the D3-induced p47^phox^ expression is enhanced with addition of RRD-251 at 48 hours, indicating a significant impact on differentiation (Figure 5B). In sum, RRD-251 augments D3-induced differentiation as measured by G_1_/_0_ cell cycle arrest, CD11b, CD14, and inducible oxidative metabolism. Figures 1-5 are summarized in Table 1.

**Table 1:** Summary table of phenotypic markers

		24h	48h	72h
**G0/G1 % cell population**				
	C	48.57	53.43	51.97
	D3	57.23	58.40	72.77
	RRD	53.60	71.00	52.47
	RRD + D3	62.57	77.23	68.90
**S % cell population**				
	C	31.87	26.50	32.20
	D3	28.00	24.50	16.87
	RRD	26.97	16.67	28.27
	RRD + D3	23.07	12.73	18.93
**G2/M % cell population**				
	C	19.53	19.90	15.77
	D3	14.80	16.90	10.37
	RRD	19.43	12.20	19.23
	RRD + D3	14.37	10.00	12.13
**Cell Growth (x 10^6)**				
	C	0.26	0.56	1.39
	D3	0.21	0.46	1.05
	RRD	0.20	0.35	0.95
	RRD + D3	0.15	0.27	0.54
**CD38 % cell population**				
	C	4.97	4.95	5
	D3	75.67	91.85	86.5
	RRD	7.90	5.25	4.5
	RRD + D3	78.87	90.7	77.67
**CD38 mean expression**				
	C	473.67	470.33	545.67
	D3	2633.67	4542.33	5540.00
	RRD	474.00	489.33	641.67
	RRD + D3	3228.00	4518.33	5018.67
**CD11b % cell population**				
	C	5.00	5.00	4.93
	D3	23.37	47.33	61.33
	RRD	4.87	8.87	7.33
	RRD + D3	27.03	70.23	67.77
**CD14 % cell population**				
	C	4.90	5.10	5.00
	D3	9.17	31.67	41.43
	RRD	3.40	14.27	14.53
	RRD + D3	16.60	48.33	53.90
**Inducible respiratory burst % cell population**				
	C			5.53
	D3			50.47
	RRD			5.80
	RRD + D3			62.87

### RRD-251 enhances D3 regulation of GSK-3 activity by c-Raf and Akt

**Figure 6:**
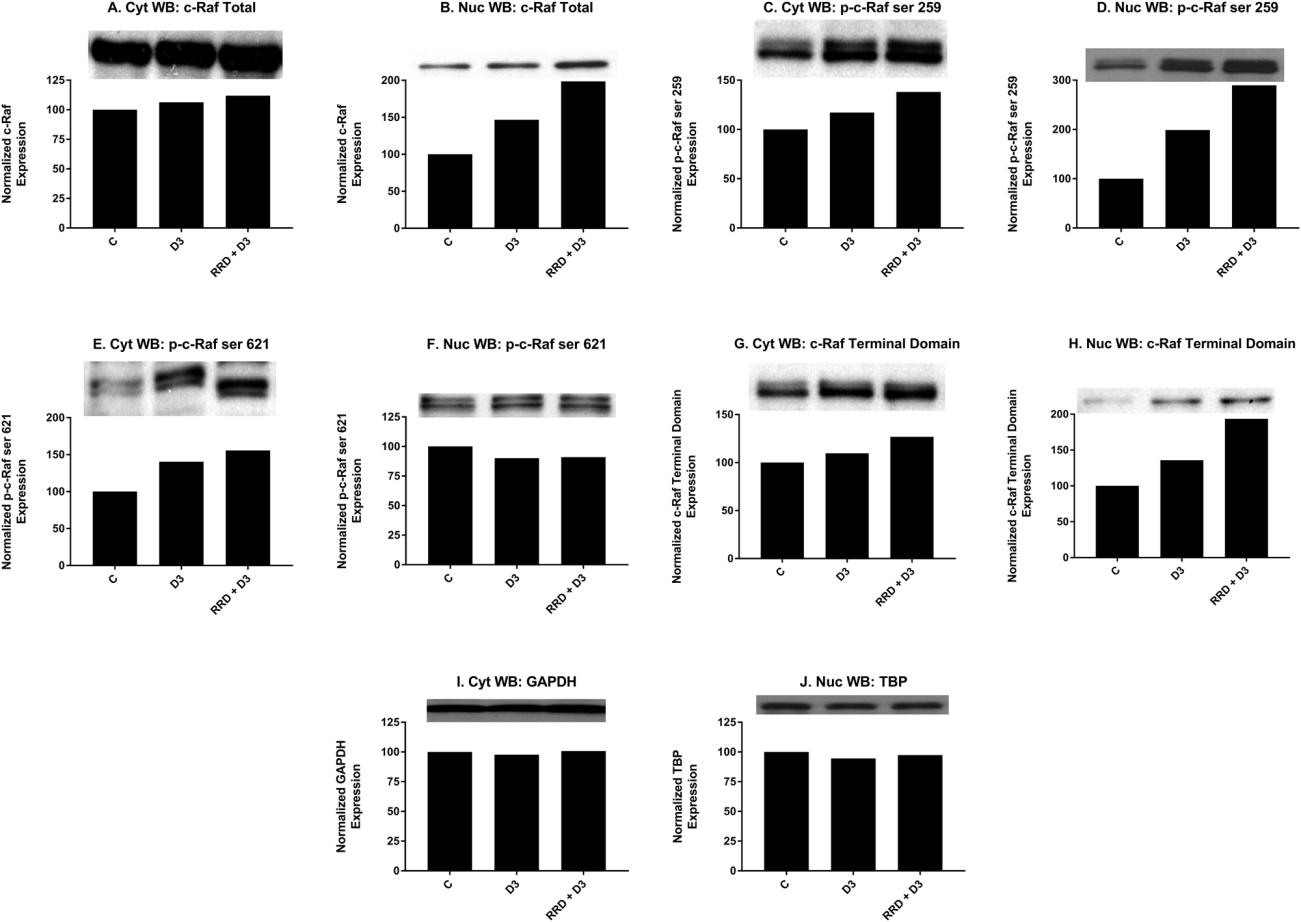
RRD-251 promotes D3-induced nuclear localization and regulation of c-Raf Changes in total expression and/or the specific phosphorylation of c-Raf were assessed by Western blotting. 25 μg of protein from cytoplasmic and nuclear lysates collected 48 hours after treatment were resolved on 12% polyacrylamide gels. **(A-B)** D3 induces the nuclear translocation of c-Raf concurrent with a small increase in cytoplasmic expression, effects which are augmented with RRD-251 co-treatment. **(C-D)** D3 promotes the phosphorylation of c-Raf at serine 259 in both cytoplasmic and nuclear samples; addition of RRD-251 enhances phosphorylation at this site in both compartments. **(E-F)** D3 promotes the phosphorylation of c-Raf at serine 621 in the cytoplasm, and RRD-251 enhances this in co-treated samples. Nuclear phosphorylation of c-Raf at serine 621 is slightly decreased, with no enhancement in co-treated samples. **(G-H)** D3 promotes the phosphorylation of the p-c-Raf CTD (ser 289/296/301) in both the cytoplasm and nucleus, phosphorylation of the c-terminal domain is augmented by RRD-251 co-treatment. **(I-J)** Loading control used: GAPDH for cytosolic proteins and TBP for nuclear proteins.

**Figure 7:**
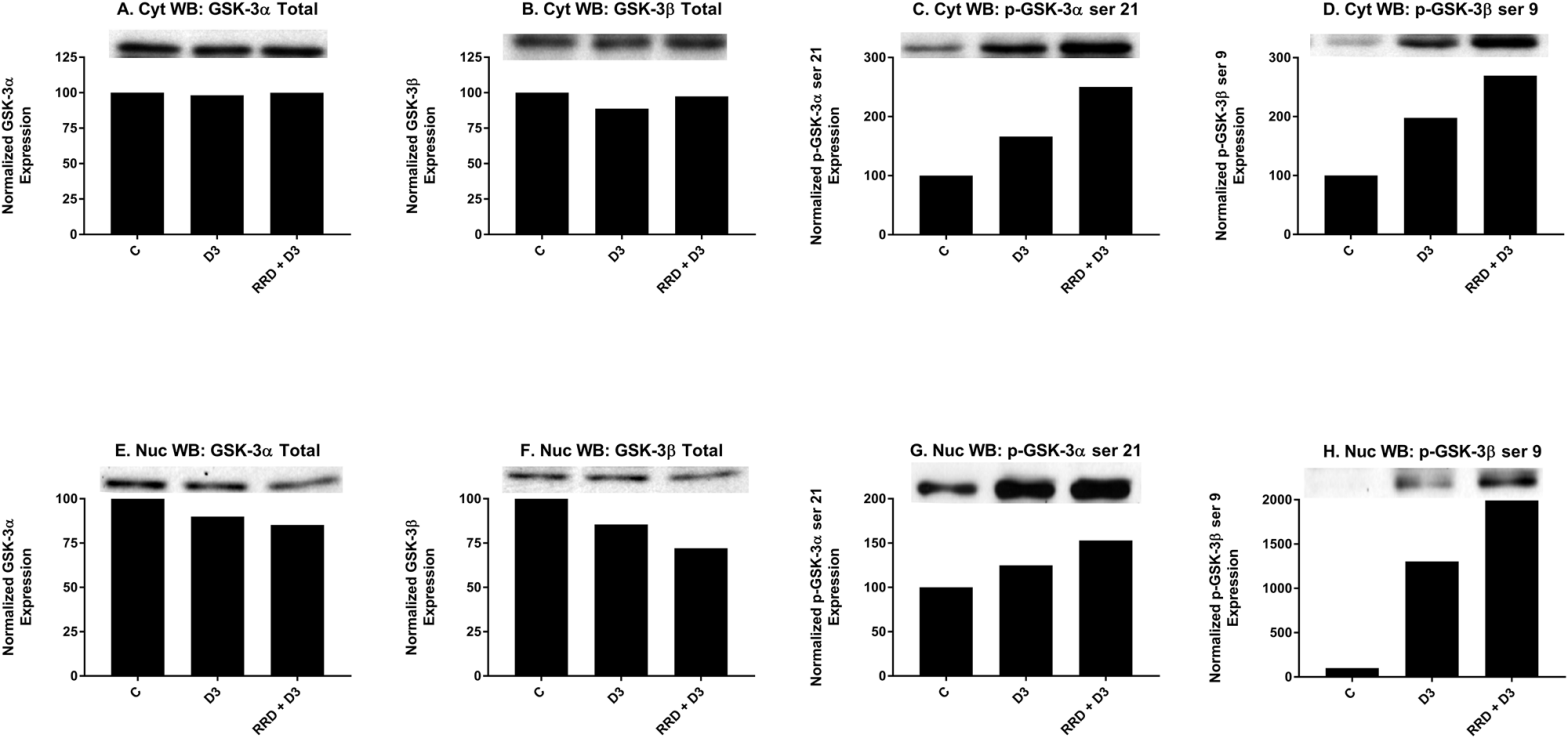
RRD-251 promotes D3-induced regulation of GSK-3 expression and inhibitory phosphorylation Changes in total expression and the specific inhibitory phosphorylation of GSK-3 α/β were assessed by Western blotting. 25 μg of protein from cytoplasmic and nuclear lysates collected 48 hours after treatment were resolved on 12% polyacrylamide gels. **(A-B)** Total cytoplasmic expression of GSK-3 α/β are unchanged with D3 or co-treatment. **(C-D)** D3 promotes cytoplasmic serine 21/9 (inhibitory) phosphorylation of GSK-3 α/β. Co-treatment with RRD-251 enhances the D3-induced phosphorylation of serine 21/9. **(E-F)** Nuclear expression of GSK-3 α/β decreases after D3 treatment, with further reductions in expression with RRD-251 co-treatment. **(G-H)** D3 promotes nuclear serine 21/9 (inhibitory) phosphorylation of GSK-3 α/β. Co-treatment with RRD-251 enhances the D3-induced phosphorylation of serine 21/9.

Motivated by the enhanced induction of nuclear c-Raf and p-GSK-3 ser 21/9 complexes with RRD-251 in RA-induced differentiation, we sought evidence of a common c-Raf/GSK-3 axis in D3-induced differentiation. Similar to RA, D3 treatment results in increased nuclear localization of c-Raf by 48 hours (Figure 6B). Consistent with increased c-Raf nuclear activation, the corresponding levels of phosphorylated nuclear p-c-Raf ser 259 (Figure 6D) and p-c-Raf C-terminal domain, aka p-c-Raf ser 289/296/301 (p-c-Raf CTD) (Figure 6H) also increased with D3 treatment. Nuclear expression of p-c-Raf ser 621 however, was not increased (Figure 6F). Additionally with D3, GSK-3 total nuclear expression modestly decreased (Figure 7E-7F), while the inhibitory phosphorylation of nuclear GSK-3 at the ser 21/9 site increased, modestly for GSK-3α (Figure 7G) and markedly for GSK-3β (Figure 7H). All of these expression changes were enhanced in magnitude with the addition of RRD-251 to D3 treatment.

**Figure 8:**
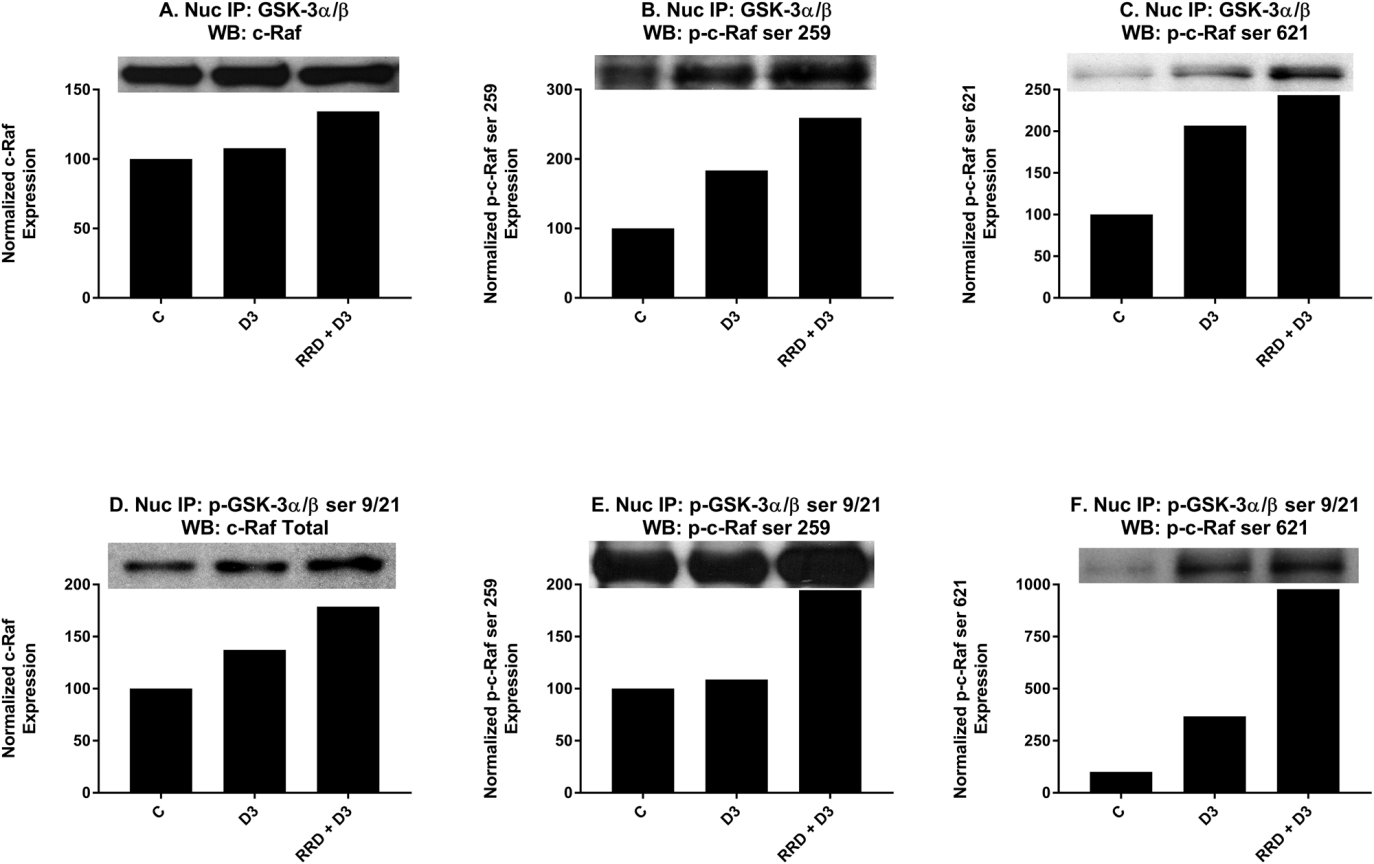
RRD-251 enhances D3-induced nuclear interactions between c-Raf and GSK-3 **(A-C)** Total GSK-3 α/β antibody was used as the precipitating antibody and the resulting blots were probed with total c-Raf, p-c-Raf ser 259, p-c-Raf ser 621, and p-c-Raf CTD (data not shown). For total c-Raf, p-c-Raf ser 259, and p-c-Raf ser 621, increased interactions were seen with D3, and RRD-251 enhanced these interactions for each case. No detectable interactions between total GSK-3 and p-c-Raf CTD were noted (data not shown). **(D-F)** A p-GSK-3α/β ser21/9 was used as the precipitating antibody and the resulting blots were probed with total c-Raf, p-c-Raf ser 259, p-c-Raf 621, and c-Raf C-terminal domain (data not shown). For total c-Raf, p-c-Raf ser259, and p-c-Raf ser621, increased interactions were seen with D3 with further increases seen with RRD-251 co-treatment. No detectable interactions between total p-GSK-3α/β ser21/9 and p-c-Raf CTD were noted (data not shown).

As the enhancement of c-Raf and GSK-3 expression changes with RRD-251 and D3 co-treatment was consistent with RRD-251 and RA co-treatment [23], we next sought to determine if like RA there was a direct interaction between c-Raf and GSK-3 for D3. Cells were treated as indicated for 48 hours and co-immunoprecipitations using nuclear lysate were performed between total GSK-3 and a panel of c-Raf (Figure 8A), p-c-Raf ser 259 (Figure 8B), p-c-Raf ser 621 (Figure 8C), and p-c-Raf CTD. With D3 treatment, there was a small increase in binding between GSK-3 and c-Raf (Figure 8A), and greater binding interactions with both p-c-Raf ser 259 (Figure 8B) and p-c-Raf ser 621 (Figure 8C). No binding between GSK-3 and p-c-Raf CTD was detected (data not shown). When repeated with p-GSK-3 ser 21/9, co-immunoprecipitations showed a greater increase in binding between p-GSK-3 ser 21/9 with c-Raf (Figure 8D) than total GSK-3, a small increased interaction with p-c-Raf ser 259 (Figure 8E), and a large increase in binding with p-c-Raf ser 621 (Figure 8F). Again, no binding with p-c-Raf CTD was detected (data not shown). Significantly, each of the interactions that were increased with D3 was enhanced with RRD-251 co-treatment.

### RRD-251 augments D3-induced GSK-3 interactions with the VDR, RXRα, and Erk1/2

**Figure 9:**
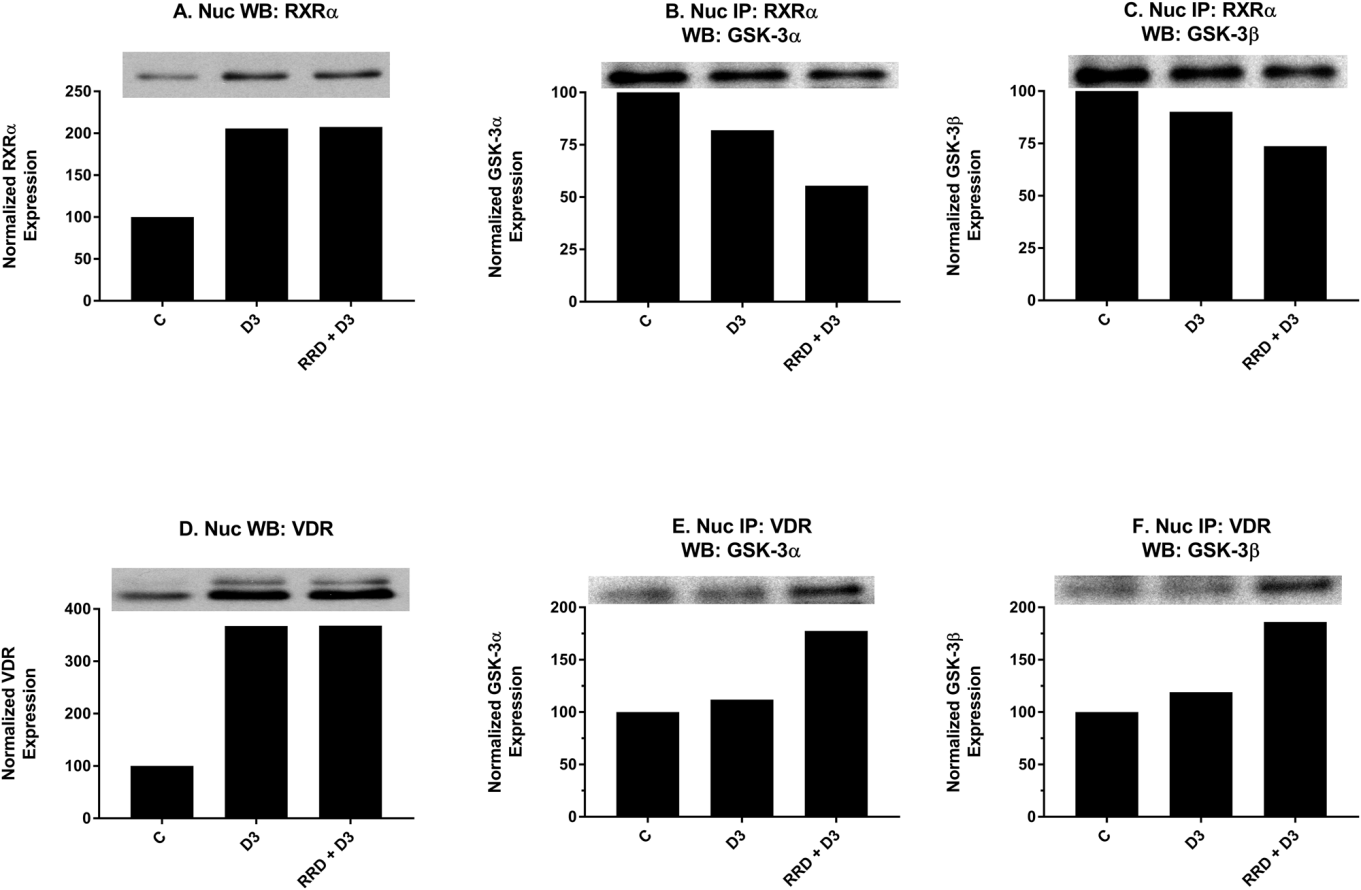
RRD-251 enhances D3-induced interactions of GSK-3 with RXR and VDR RXRα and VDR nuclear expression were assessed by Western blotting, and nuclear interactions with GSK-3 were assessed by immunoprecipitation. Nuclear lysates collected 48 hours after treatment and were resolved in 12% polyacrylamide gels. 25 μg of protein was loaded per well for Western blotting. **(A)** D3 promotes increased nuclear expression of RXRα with no further enhancement in RRD-251 co-treated samples. **(B-C)** An RXRα antibody was used as the precipitating antibody and the resulting blot was probed with total GSK-3α/β. D3 promotes a decrease in interaction between RXRα and GSK-3α/β. RRD-251 co-treatment further diminishes the interaction. **(D)** D3 promotes increased nuclear expression of VDR with no further enhancement in RRD-251 co-treated samples. **(E-F)** A VDR antibody was used as the precipitating antibody and the resulting blot was probed with GSK-3α/β. D3 alone promotes only a small increase in interactions between VDR and GSK-3α/β, which is much augmented by co-treatment.

After confirming that the c-Raf/GSK-3 axis was activated by D3 and further enhanced with RRD-251, effects on downstream interactions between GSK-3 and transcriptional regulators for monocytic differentiation were explored. Nuclear expression of RXRα (Figure 9A) and VDR (Figure 9D), two transcriptional regulators which heterodimerize to activate transcription of genes with promoters containing VDREs, increases with D3-treatment but has no further enhancement with RRD-251 (Figure 9A and 9D). To explore whether GSK-3 might modulate RXRα and VDR activity rather than total expression, nuclear co-immunoprecipitations were performed. Similar to RA’s effect of decreasing GSK-3 and RARα interactions [23], and despite increased RXRα expression overall (Figure 9A), D3 decreased this novel interaction between total GSK-3 and RXRα (Figure 9B and 9C). In contrast, D3 instead promotes a novel small increase in binding between total GSK-3 and VDR (Figure 9E and 9F) which may reflect a fundamental difference in GSK-3’s regulation of RARα and VDR. Importantly, the addition of RRD-251 enhances the effect of D3 on GSK-3 interactions with RXR and VDR, which is consistent with the notion that the changes contribute to differentiation.

**Figure 10:**
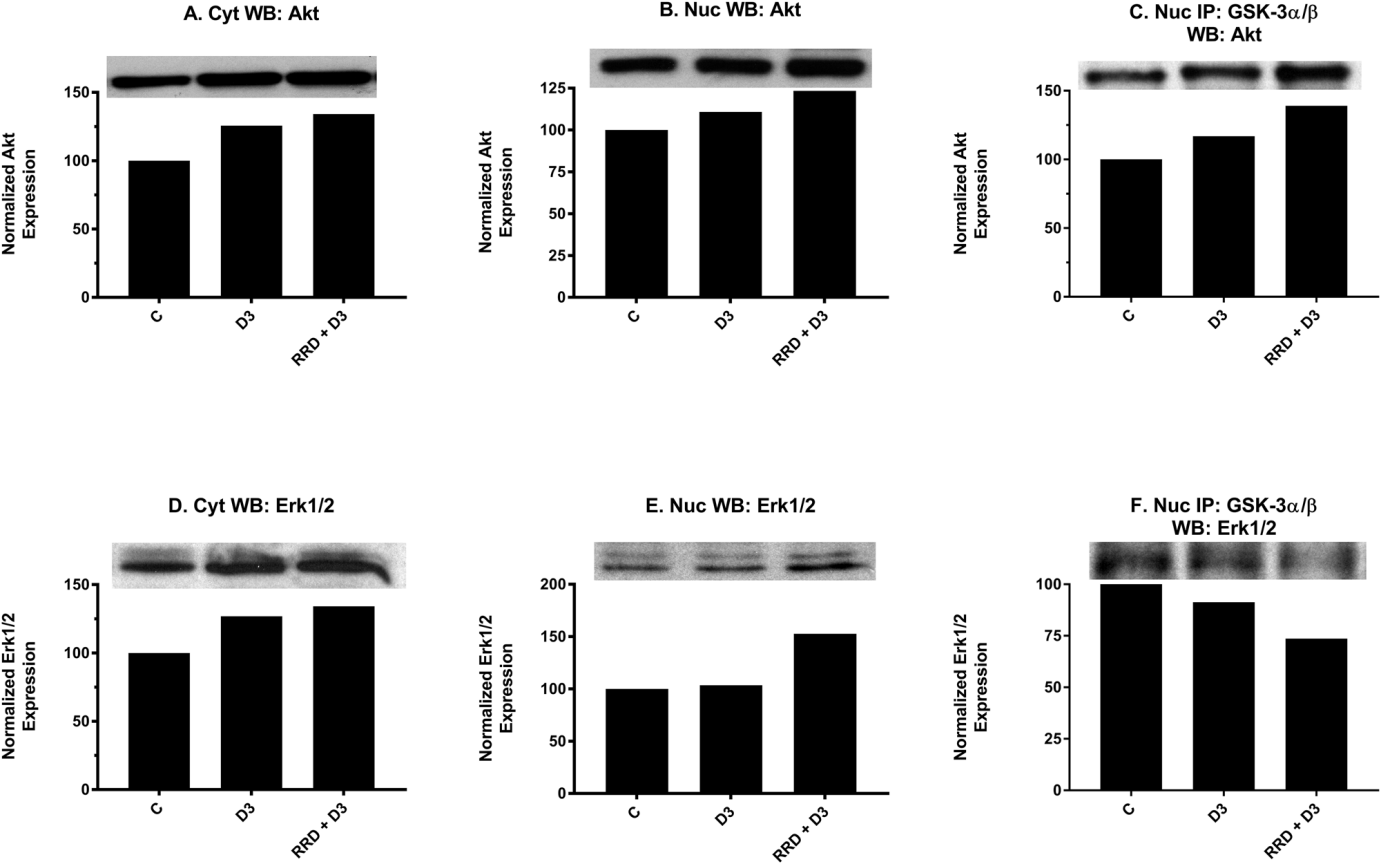
RRD-251 augments D3-induced Akt and Erk1/2 nuclear interactions with GSK-3 Akt and Erk1/2 cytoplasmic and nuclear expression were assessed by Western blotting, and nuclear interactions with GSK-3 were assessed by immunoprecipitation. Nuclear lysates collected 48 hours after treatment and were resolved in 12% polyacrylamide gels. 25 μg of protein was loaded per well for Western blotting. 250 μg of pre-cleared nuclear lysate collected at 48 hours post treatment was incubated overnight with 3.5 μg of the precipitating antibody with magnetic beads and resolved on 12% polyacrylamide gels. **(A-B)** D3 induces a small increase in both cytoplasmic and nuclear expression of total Akt, which is augmented by RRD-251 co-treatment. **(C)** A total GSK-3α/β antibody was used as the precipitating antibody and the resulting blot was probed with total Akt. D3 promotes an increased interaction with Akt, which is enhanced with the addition of RRD-251. **(D-E)** D3 promotes an increase in cytoplasmic but not nuclear expression of Erk1/2. Combined treatment with RRD-251 promotes increases in both cytoplasmic and nuclear Erk1/2. **(F)** A GSK-3α/β antibody was used as the precipitating antibody and the resulting blot was probed with Erk1/2. D3 reduces the interaction between GSK-3α/β and Erk1/2, addition of RRD-251 further diminishes the interaction.

To further probe the attributes of GSK-3 inhibition, its interaction with Akt, a classical inhibitor of GSK-3, in the nucleus was examined. At 48 hours, nuclear expression of Akt (Figure 10B) mildly increases with D3 treatment and is enhanced with RRD-251. Co-immunopreciptation assays indicate that binding between total GSK-3 and Akt is promoted with D3 and further potentiated by RRD-251 (Figure 10C). Akt may ergo collaborate to inhibit GSK-3 downstream of D3, and RRD-251 enhances this.

Motivated by the reduced interactions between GSK-3 and Erk1/2 observed with RA and RRD-251 [23], we looked to determine whether this was another conserved feature for D3-induced differentiation. Nuclear expression of total Erk1/2 remained stable with D3 treatment; however, levels increased with RRD-251 co-treatment (Figure 10E). Despite total expression increasing, the interaction between total GSK-3 and total Erk1/2 decreases with D3 and decreases further with addition of RRD-251 (Figure 10F). RRD-251 causes increasing nuclear Erk1/2 expression and diminishing interaction with GSK-3 which may result in an expanded nuclear pool of free Erk1/2 able to amplify pro-differentiation signals via its other nuclear partners.

### RRD-251 enhances D3-induced Vav1 interaction with Erk1/2

**Figure 11:**
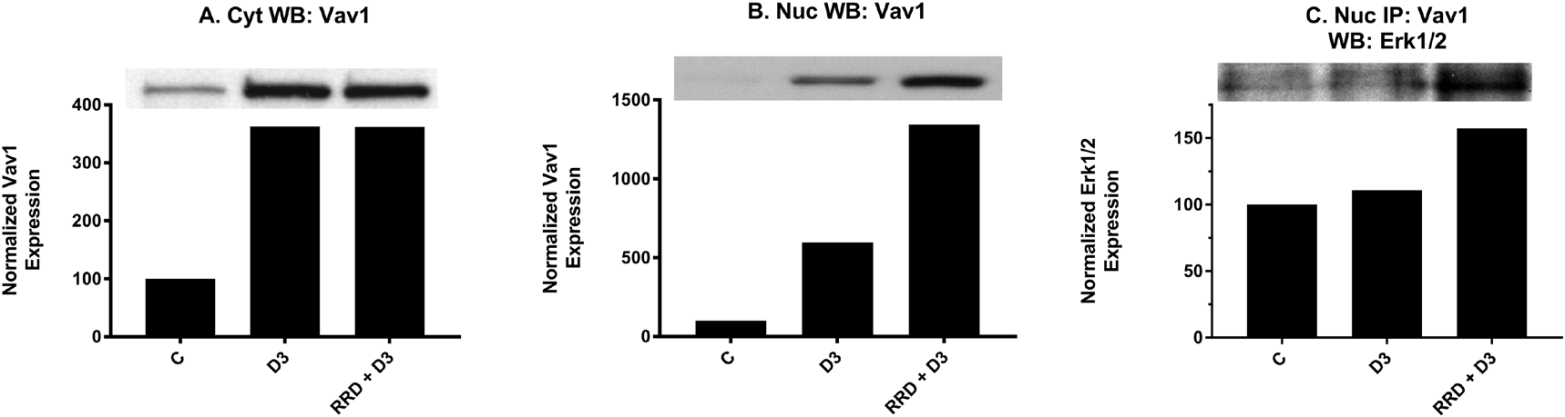
RRD-251 increases the D3-induced Erk1/2-Vav1 nuclear interaction Vav1 cytoplasmic and nuclear expression was assessed by Western blotting and nuclear interactions with Erk1/2 were assessed by immunoprecipitation. Cytoplasmic and nuclear lysates collected 48 hours after treatment and were resolved in 12% polyacrylamide gels. 25 μg of protein was loaded per well for Western blotting. **(A-B)** D3 promotes an increase in both cytoplasmic and nuclear expression of Vav1. Cytoplasmic and nuclear modulation of Vav1 expression by RRD-251 co-treatment differ, as there is no further cytoplasmic enhancement of Vav1 expression, yet nuclear expression is greatly enhanced by co-treatment. **(C)** For immunoprecipitation, 250 μg of pre-cleared nuclear lysates collected after 48 hours post treatment were incubated overnight with 3.5 μg of the precipitating antibody with magnetic beads and resolved on 12% polyacrylamide gels. A Vav1 antibody was used as the precipitating antibody and the resulting blot was probed with Erk1/2. D3 alone promotes an increase in interaction between Vav1 and Erk1/2, and addition of RRD-251 enhances the increase.

We next examined if an increased nuclear pool of Erk1/2 might interact with nuclear targets for enhancing differentiation. Vav1 presented as a potential target. The nuclear expression of Vav1 increased at 48 hours with D3, and RRD-251 dramatically amplified the effect (Figure 11B). Interaction between Vav1 and Erk in the nucleus increases slightly with D3, and RRD-251 augments the increase (Figure 11C). The interaction between Erk1/2 and Vav1 is known to be required for expression of the CD11b differentiation marker as Vav1 must bind the CD11b promoter for its transcriptional activation [39]. As reported above, D3-induced expression of CD11b, as measured by flow cytometry, is significantly increased when combined with RRD-251 at 48 hours. The data are thus consistent with the notion that increased nuclear Erk1/2 can contribute to differentiation.

## DISCUSSION

Differentiation therapy is highly successful for APL where retinoic acid induces a high remission rate. Retinoic acid and vitamin D use a significant number of common signaling pathways. Moreover, epidemiologic studies showed that high vitamin D levels are associated with reduced cancer risks [40]. Unfortunately, the clinical utility of vitamin D in differentiation therapy for leukemia has been hampered by vitamin D-induced hypercalcemia due to the therapeutic levels of vitamin D. In the present work we evaluated if RRD-251 is able to enhance vitamin D3-induced differentiation of leukemic cells, as a proof of principle that intervention in c-Raf- associated pathways using combination therapy can be more effective than D3 alone.

The present studies show that the small molecule RRD-251 enhances D3-induced signaling seminal to differentiation of human myeloid leukemia HL-60 cells and subsequent monocytic differentiation. RRD-251 enhanced the D3-induced increase in the amount of c-Raf in the nucleus. That increased c-Raf included its p-CTD and ser 259 forms. In the nucleus p-c-Raf ser 259 and p-c-Raf ser 621 bound GSK-3. There was increased GSK-3 phosphorylated at its ser 21/9 site. Phospho-ser 21/9 are known to be inhibitory sites on GSK-3. Associated with this was enhanced Akt expression (consistent with previous reports showing that siRNA KD of Akt diminishes RA-induced differentiation assessed by CD11b [41]) and enhanced Akt binding to GSK-3. Akt is also known as a GSK-3 inhibitor [42–44], suggesting a cascade of collaborating inhibitory effects on GSK-3 precipitated by c-Raf. GSK-3 binding to VDR was enhanced. GSK-3 inhibition is known to increase VDR transcriptional activity [26]. Coincidentally GSK-3 binding to Erk1/2 also diminished and Erk1/2 binding to Vav1 increased, where Vav1 and Erk1/2 are both known to contribute propulsion to differentiation, suggesting that the c-Raf-GSK-3 interaction may be a nexus originating pathways driving differentiation. The subsequent D3-induced cell differentiation and G_0_ cell cycle arrest was enhanced. In sum the data motivate a paradigm whereby a c-Raf/GSK-3/VDR axis drives differentiation and signaling along this axis is promoted by RRD-251.

It is potentially noteworthy that the involvement of Akt and Erk1/2 in this D3 driven pathway for differentiation provides an obvious mechanism of signal integration with peptide hormone/ growth factor signaling. In the case of Akt, for example, receptor tyrosine kinase signaling activating PI3K to phosphorylate PIP2 to PIP3 making membrane domains to aggregate PDK and Akt resulting in Akt activation is a classical paradigm for growth factor signaling. In the case of Erk1/2, growth factor signaling through various membrane receptors, such as EGFR or PDGFR, results in activation of the c-Raf/Mek/Erk axis and Erk1/2 nuclear translocation to phosphorylate and activate transcription factors. Hence a simple ramification of the D3 signaling paradigm proposed here is that the D3-induced differentiation pathway and growth pathways - such as in self-renewal of stem like cells - can interact to potentially enhance, eg. when one activates a component used by the other, or mute, eg. when one competes for components with the other, each other.

**Figure 12:**
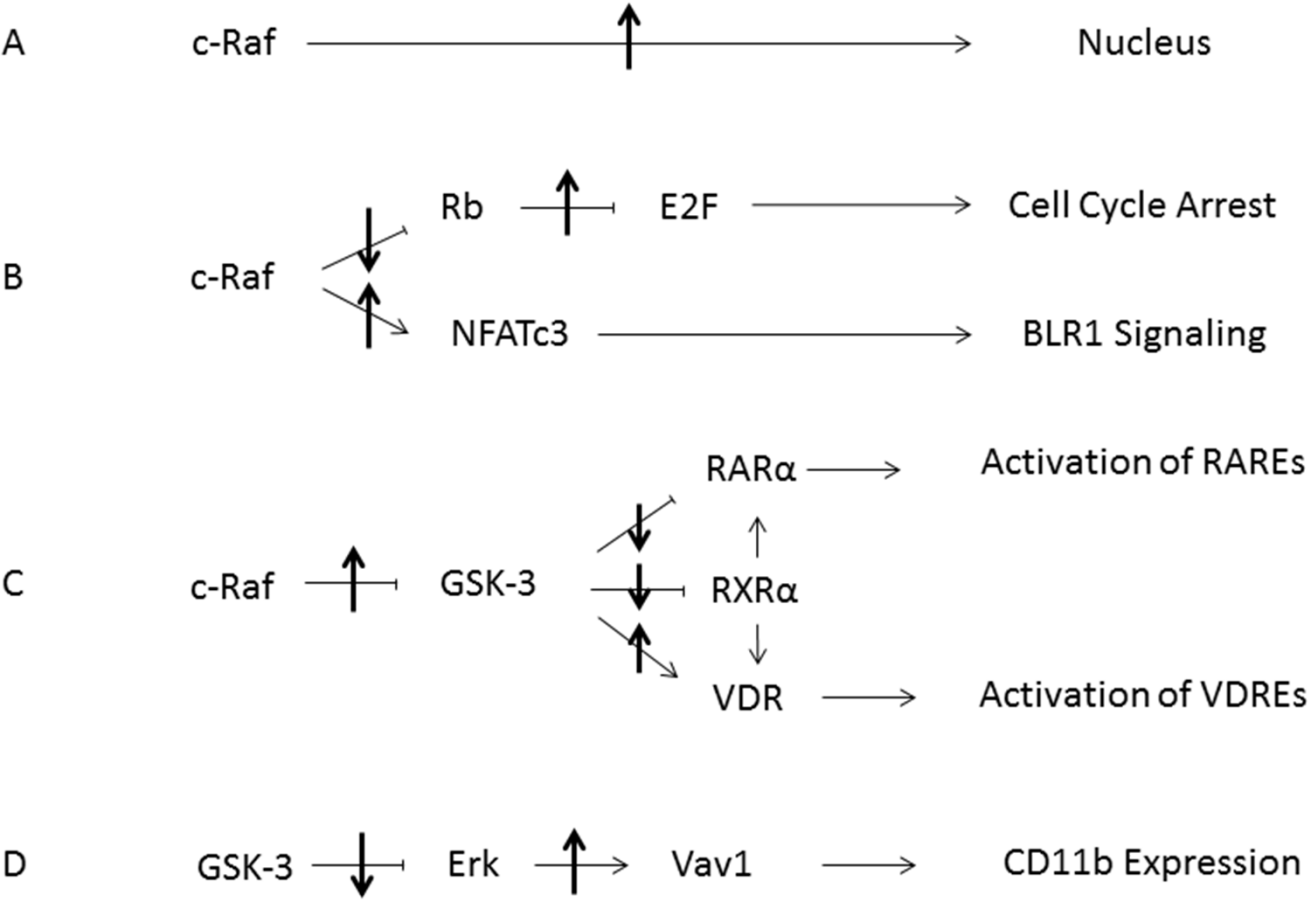
Summary Diagram of RRD-251 Modulation of RA/D3 Differentiation **(A)** Increased nuclear translocation of c-Raf is induced by RRD-251. **(B)** Inhibition of the Retinoblastoma protein by nuclear c-Raf is relieved, allowing for increased E2F sequestration and cell cycle arrest. The freed nuclear c-Raf then activates NFATc3, which binds the promoter for BLR1/CXCR5. **(C)** The increased pool of nuclear c-Raf inhibits GSK-3 thereby relieving inhibition of RARα and RXRα, increasing interactions with the VDR, which enhances transcriptional activation of RAREs and VDREs respectively. **(D)** Inhibition of GSK-3 leads to decreased inhibitory interactions with Erk. The increase in free Erk then interacts with Vav1, which in turn binds the promoter for CD11b. Up and down arrows indicate effect of RRD-251 on these processes.

The effects seen here are related to those observed with retinoic acid (RA)-induced differentiation of HL-60 cells, where a c-Raf/GSK-3/RARα axis was invoked. In the case of RA, it was found that RA caused formation of a cytosolic signalsome susceptible to plasma membrane originated activation that caused the nuclear translocation of c-Raf which in addition to GSK-3 bound transcription factors to enable RARα transcriptional activation as well as being sequestered by pS608 phosphorylated RB [23]. Hypophosphorylation of RB with G_1/0_ arrest would dissociate c-Raf from RB and contribute to progression of differentiation. Interestingly this may be a mechanistic rationale for the proverbial classical prejudice for association of differentiation and proliferative arrest. In the case of D3 c-Raf/GSK-3 also appears to perform an enabling function, in this case for VDR versus RARα. The process of RA or D3-induced differentiation in these myelomonocytic progenitor cells segregates into a pre-commitment phase that is not lineage specific anteceding a later phase where commitment to lineage occurs. c-Raf nuclear translocation is a novel feature of c-Raf kinase function that we reported [17, 18] and appears to be at least a partial mechanistic rationalization for the occurrence of a precommitment phase that is lineage agnostic. The picture that is emerging of the role of nuclear disposition of c-Raf is to function as an enabler for transcriptional activation by RARs or VDRs either by phosphorylation of co-regulatory transcription factors at the promoter such as for NFATc3 or phosphorylation of GSK-3 to regulate RARα or VDR phosphorylation and ergo transcriptional activity, all of which activity is connected to pS608 RB which sequesters nuclear c-Raf such that hypophosphorylation - as in cell cycle arrest - would release it. Here we have pharmacologically provoked that release using a small molecule, RRD-251. Figure 12 is a schematic summary of the effects of RRD-251 on processes that propel differentiation.

## MATERIALS AND METHODS

### Cell cultures and treatments

The HL-60 human myeloblastic leukemia cells were originally an early pass of cells derived from the original patient samples, generously gifted by Dr. Robert Gallagher and continuously maintained in this laboratory as previously published [23]. The cells used were certified as mycoplasma free HL-60 by Bio-Synthesis, Lewisville, TX, in August 2017. The HL-60 cells were grown in RPMI 1640 (Invitrogen, Carlsbad, CA) supplemented with 5% fetal bovine serum (Hyclone, Logan, UT) and 1x antibiotic/antimycotic (Sigma, St. Louis, MO) in a 5% CO2 humidified atmosphere at 37°C. Cells were continuously cultured in the constant exponential growth phase, not exceeding 2.0 x10^6^, with cell viability exceeding 95% by 0.2% trypan blue (Invitrogen, Carlsbad, CA) exclusion dye via hemocytometry. Experimental cultures were initiated at a density of either 0.1 x 10^6^ for 24, 48, and 72 hour time points for flow cytometry, or 0.2 x 10^6^ for 8 hour flow cytometry time points and 48 hour lysate collection. All cells and lysate collected used the same treatment conditions of 0 μM or 10 μM RRD-251 Hydrochloride (Sigma) and 0 μM or 0.5 μM 1,25-dihydroxyvitamin D3 (Cayman, Ann Arbor, MI). Cells were harvested at 8, 24, 48, and 72 hours post-treatment. Three biological replicates of each experiment were performed unless stated otherwise.

### CD11b/CD14/CD38 expression studies by flow cytometry

Live HL-60 cells (0.5 x 10^6^) were harvested by centrifugation at 120 x g for 5 minutes and the pellet was resuspended in 200 μL of PBS containing 2.5 μL of allophycocyanin-conjugated anti-body, phycoerythrin-conjugated CD14 anti-body, or phycoerythrin-conjugated CD38 antibody (BD Biosciences). After a 1 hour incubation period at in a humidified atmosphere of 5% CO_2_ at 37°C, cells were analyzed by flow cytometry (LSRII flow cytometer, BD Biosciences) using 633-nm red excitation laser and 660/20 band pass filter (APC) or 488-nm blue excitation laser and 576/26 band pass filter (PE). Exclusion gates were set to 95% of control cells to determine the percent of positive cells. For determination of the relative CD38 expression per cell, median values of the fluorescence histogram were derived, and shifts in relative median expression levels were used to determine the effect of RRD-251 on CD38 expression [23].

### Measurement of respiratory burst (inducible oxidative metabolism)

0.5 x 10^6^ HL-60 cells were collected by centrifugation and resuspended in 200 μL of PBS containing 10 μM 5-(and-6)-chloromethyl-2’,7’-dichlorodihydrofluorescein diacetate acetyl ester (H2-DCF, Molecular Probes, Eugene, OR) and 0.4 μg/mL 12-O-tetradecanoylphorbol-13-acetate (TPA, Sigma) then incubated for 20 minutes at 37°C. Flow cytometric analysis was performed (BD LSRII flow cytometer) using 488-nm laser excitation and emission collected through 505 long-pass dichroic and 530/30 band-pass filters. Exclusion gates were set to 95% of control cell that were not treated with TPA. The shift in fluorescence intensity in response to TPA was used to determine the percent of cells capable of generating inducible superoxide, a characteristic of mature, differentiated cells. Cells that have not been treated with or without TPA and D3-treated cells without TPA typically showed indistinguishable DCF fluorescence histograms [45].

### Cell cycle analysis

HL-60 cells (0.5 x 10^6^) were harvested by centrifugation and resuspended in 200 μL cold hypotonic propidium iodide (PI) staining solution containing 50 μg/mL propidium iodide, 1 μL/mL Triton X-100 and 1 mg/mL sodium citrate. Cells were incubated overnight at 4° C and analyzed by flow cytometry (BD LSRII) using 488-nm excitation and collected through 550 long-pass dichroic and 576/26 band-pass filters. Doublets were identified and excluded from analysis using a PI signal width versus area plot [23].

### Western blotting and immunoprecipitation

Cytoplasmic and nuclear cellular fractionation was done with the NE-PER kit (Pierce Thermo Scientific, Lafayette, CO), following the manufacturer’s instructions with the addition of protease and phosphatase inhibitors. All lysates were stored at -80° C until use. After lysate collection, cellular debris and membranes were pelleted by centrifugation at 16060 x g for at least 10 minutes, and the BCA assay (Pierce Thermo Scientific, Lafayette, CO) was performed to quantify protein concentrations. 250 μg of protein was pre-cleared with PureProteome Protein G magnetic beads (Millipore, Billerica, MA) for at least 2 hours, and then incubated overnight with 3.5 μg of primary antibody and fresh magnetic beads. The beads were washed 3 times, boiled for 10 minutes, and dissociated proteins were resolved by SDS/PAGE analysis, followed by transfer to polyvinylidene fluoride (PVDF) membrane (Millipore, Billerica, MA). For Western blotting, 25 μg of protein was resolved on 12% polyacrylamide gel, followed by electrotransfer for 1 hour at 400mA. Membranes were then blocked in milk for at least 1 hour before overnight incubation at 4°C in primary antibody. c-Cbl (C-15) antibody was from Santa Cruz Biotechnology (Santa Cruz, CA). c-Raf antibody was from BD Biosciences (San Jose, CA). GSK-3 antibody was from Sigma Aldrich (St. Louis, MO). Erk1/2 and p-c-Raf ser 621 antibodies were from Pierce Thermo Scientific (Lafayette, CO). Akt, GAPDH, GSK-3, p47^phox^, p-c-Raf ser 259, p-c-Raf CTD, p-GSK-3 ser 21/9, TBP, Vav1, VDR, RXRα, horseradish peroxidase anti-mouse and horseradish peroxidase anti-rabbit were from Cell Signaling (Danvers, MA). Enhanced chemiluminescence ECL reagent (GE Healthcare, Pittsburg, PA) was used for detection.

### Statistics

To determine statistical significance of results, one-way ANOVA tests using Bonferroni’s multiple comparisons test comparing each sample against control or D3. Significance threshold was set at p<0.05. There were 3 repeats for each time point of CD38 (8, 24, 48, 72 hours), CD11b, CD14, cell growth, and cell cycle analysis (24, 48, 72 hours), and ROS production (72 hours) [23].
